# Morphological, physiological, and molecular scion traits are determinant for salt-stress tolerance of grafted citrus plants

**DOI:** 10.3389/fpls.2023.1145625

**Published:** 2023-04-20

**Authors:** Vicente Vives-Peris, María F. López-Climent, María Moliner-Sabater, Aurelio Gómez-Cadenas, Rosa M. Pérez-Clemente

**Affiliations:** Department of Biology, Biochemistry and Natural Sciences, Universitat Jaume I, Castelló de la Plana, Spain

**Keywords:** abiotic stress, grafting, photosynthesis, phytohormones, salinity, vacuole

## Abstract

**Introduction:**

Citrus productivity has been decreasing in the last decade in the Mediterranean basin as a consequence of climate change and the high levels of salinity found in the aquifers. Citrus varieties are cultivated grafted onto a rootstock, which has been reported as responsible for plant tolerance to adverse situations. However, other important factors for stress tolerance relying in the scion have been less studied. The aim of this study was to evaluate the effect of the grafted scion on citrus tolerance to salt stress.

**Methods:**

Four different citrus rootstock/scion combinations were subjected to salt stress for 30 days, using Carrizo citrange (CC) or *Citrus macrophylla* (CM) as rootstocks, and Navelina orange (NA) or Oronules mandarin (OR) as scions. CM-OR was the most tolerant combination, whereas CC-NA was the most sensitive one.

**Results and discussion:**

Our results support the idea that the rootstock plays an important role in salt stress tolerance, but scion is also crucial. Thus, photosynthesis and transpiration, processes regulated by abscisic acid and jasmonic acid, are determinant of plant performance. These photosynthetic parameters were not affected in plants of the salt-tolerant combination CM-OR, probably due to the lower intoxication with Cl^−^ ions, allowing a better performance of the photosynthetic machinery under stress conditions. The different stomatal density of the two citrus scions used in this work (higher in the sensitive NA in comparison to the tolerant OR) also contributes to the different tolerance of the grafted plants to this adverse condition. Additionally, *CsDTX35.1* and *CsDTX35.2*, genes codifying for Cl^−^ tonoplast transporters, were exclusively overexpressed in plants of the salt-tolerant combination CM-OR, suggesting that these transporters involved in Cl^−^ compartmentalization could be crucial for salt stress tolerance. It is concluded that to improve citrus tolerance to high salinity, it is important that scions have a versatile photosynthetic system, an adequate stomatal density, and a proper modulation of genes coding for Cl^−^ transporters in the tonoplast.

## 1 Introduction

Salt stress is one of the major environmental constrains affecting agriculture, whose incidence has increased in the last decades due to aquifer overexploitations and seawater intrusion, threatening agriculture sustainability (İbrahimova et al., 2021). Recent studies have concluded that approximately 1 billion hectares of soil are salinized worldwide (Ivushkin et al., 2019), which comprises 20% of the total cultivated and 33% of irrigated soils for agriculture (Shrivastava and Kumar, 2015). Furthermore, it is expected that this percentage dramatically increased during the 21st century as a consequence of the increase in world population and the climate change (Hassani et al., 2021).

High salinity is a combination of osmotic and ion toxic components. At initial stages of salt stress, there is an osmotic effect caused by the accumulation of sodium (Na^+^) and chloride (Cl^−^) ions in the soil, which reduces soil water potential and subsequently challenges water and nutrient uptake (Acosta-Motos et al., 2017). This adverse effect can be countered by the plant following different strategies, including accumulation of compatible osmolytes, such as proline, trehalose, or glycine-betaine (Baggett et al., 2021). The toxic component is linked to the uptake of water with excess of Na^+^ or Cl^−^ ions. The excess of Na^+^ and Cl^−^ contributes to enzyme inactivation and macromolecule disruption (Kamran et al., 2020). To mitigate this part of salt stress, plants have developed different strategies that lead to plant tolerance, such as (i) the maintenance of high K^+^/Na^+^ ratios through the exclusion of Na^+^ uptake; (ii) the dilution of Na^+^ and/or Cl^−^ ions through their translocation to the canopy; (iii) the control of transpiration, limiting the entrance of salinized water; and/or (iv) the compartmentalization of the Na^+^ and Cl^−^ ion excess in the vacuoles through tonoplast Na^+^/H^+^ antiporters (Kamran et al., 2020).

The main mechanisms to cope with salinity in citrus include (i) stomatal closure (López-Climent et al., 2008); (ii) proline accumulation (Vives-Peris et al., 2018a); (iii) alterations in the phytohormone concentrations, which play an essential role in the signaling response to stress (Vives-Peris et al., 2018b); and (iv) changes in expression of genes involved in salt stress tolerance, such as *Na^+^/H^+^ eXchanger* (*NHX*) and *High Affinity K^+^ Transporter* (*HKT*) that encode for tonoplast Na^+^ transporters responsible for their sequestration in the vacuoles (Martínez-Alcántara et al., 2015). However, with Cl^−^ excess being the most toxic component of salt stress in citrus (López-Climent et al., 2008), it seems necessary to study the behavior of its tonoplast transporters. Among them, the *ChLoride Channel* (*CLC*), *ATP-Binding Cassette* (*ABC*), and *Multidrug And Toxin Extrusion* (*MATE)* families contain members (*CLCa*, *CLCc*, *Aluminum-activated Malate Transporter 9* [*ALMT9*], or *DeToXifyng efflux carriers 33* and *35* [*DTX33*, and *DTX35*]) that have been reported as Cl^−^ transporters in the vacuole membrane, responsible for the compartmentalization of this element in the vacuole (Baetz et al., 2016; Zhang et al., 2017).

Among other regions, citrus are widely cultivated in the Mediterranean basin with scarce precipitations that force to use additional water supplies, usually with underground water that often contains high levels of ions (Ziogas et al., 2021). This fact, along with the low tolerance of citrus plants to salinity (Prior et al., 2007), leads to important reductions of their productivity (Ziogas et al., 2021). To mitigate the deleterious effects of salinity on citrus, several cultural practices have been traditionally proposed, including (i) the use of tolerant rootstocks as Cleopatra mandarin or *Citrus macrophylla* (CM) instead of the sensitive Carrizo citrange (CC) (López-Climent et al., 2008; Vives-Peris et al., 2017); (ii) the irrigation management, watering the plants with additional volume to hinder the formation of a salt bulk around roots (Bello et al., 2021); and/or (iii) the use of beneficial microorganisms (Vives-Peris et al., 2020). However, selecting appropriate tolerant scions that could cope with the adverse effect of salt stress and maintain crop performance and yield has not been usually considered. This is in part due to the lack of information on the physiological and molecular processes beyond their different scion tolerance (Ben Yahmed et al., 2016; Brito et al., 2021).

The main objective of this work was to elucidate the role of the grafted scion on citrus tolerance to salt stress. For this purpose, an experiment with four rootstock/scion combinations was conducted subjecting plants to salt stress conditions. Results have revealed the importance of different morphological, physiological, and molecular traits from the scion in the tolerance of the rootstock/scion combination to adverse soil-dependent environmental conditions, such as high salinity or drought. Thus, leaf stomatal density and the regulation of the stomatal closure seem to play an essential role since these parameters highly influence transpiration and, consequently, water and ion uptake. Additionally, Cl^−^ tonoplast channels’ *CsDTX35.1* and *CsDTX35.2* overexpression has been detected exclusively in tolerant scions, suggesting that tolerant plants could improve the compartmentalization of Na^+^ or Cl^−^ ions.

## 2 Materials and methods

### 2.1 Plant material and treatments

Two-year-old certified Navelina orange (NA; *Citrus sinensis* L. Osbeck) or Oronules mandarin (OR; *Citrus clementina* Hort. Ex Tan.) grafted onto the rootstocks Carrizo citrange (CC; *Citrus sinensis* L. Osbeck x *Poncirus trifoliata* L. Raf.), or *Citrus macrophylla* Wester (CM), having similar size and development, was used as plant material. The four different rootstock/scion combinations obtained were (1) Carrizo citrange–Navelina (CC-NA), (2) Carrizo citrange–Oronules (CC-OR), (3) *Citrus macrophylla*–Navelina (CM-NA), and (4) *Citrus macrophylla*–Oronules (CM-OR). Certified plants were provided by an authorized plant nursery (Beniplant, Peniscola, Castelló, Spain). Plant material was grown in 2.5-L black PVC pots containing peat moss as substrate under greenhouse conditions (22/30 ± 2°C day/night, 50%–70% Hr, and natural photoperiod), and watered with half-strength Hoagland solution (Manzi et al., 2015). Plants were acclimated for 2 months previously to stress imposition. The experiments were performed in the greenhouses of the Universitat Jaume I (Castelló de la Plana, Castelló, Spain; 39.991774N, 0.071120W) during the months of May to July of 2021. Following the Spanish legislation, certified plants were used in the experiments. No additional legislation applies to these plant species and the used methodologies.

Salt stress was applied by increasing the sodium chloride (NaCl) concentration in the irrigation solution to 90 mM. Plants were watered three times per week at field capacity. Plants watered without NaCl were added as controls. Leaf and root tissue samples were collected at 30 days after stress imposition. Both tissues were immediately frozen with liquid N_2_ and maintained at −80°C for further analyses (**Supplementary Figure 1**). The experiment was replicated three times with five plants per group and replicate. The analytical determinations described below were performed with three replicates from each biological sample.

### 2.2 Malondialdehyde determination

Malondialdehyde (MDA) was spectrophotometrically quantified with the methodology described by Hodges et al. (1999) with some modifications. Briefly, 200 mg of frozen material, ground to fine powder, was extracted in 2 ml of 80% ethanol by 30 min of sonication (Elma S30, Elmasonic, Elma Schmidbauer GmbH, Singen, Germany). After this, samples were centrifuged for 20 min at 4,500 rpm, and 800 μl of the supernatant was mixed with 20% trichloroacetic acid (Merck, Darmstadt, Germany) or a mix of 20% trichloroacetic acid with 0.5 thiobarbituric acid (Merck, Darmstadt, Germany). Samples were incubated in a bath at 90°C for 1 h, and cooled down in ice for 10 min. Finally, samples were centrifuged and measured with a spectrophotometer (Spectronic Genesys 10 UV, Thermo, Waltham, MA, USA) at 440, 532, and 600 nm. The concentration of MDA content was achieved following the calculations described in Arbona et al. (2008).

### 2.3 Chloride and sodium analysis

The analysis of total Cl^−^ ion content was performed in leaf and root tissues by automatic titration with a chloride analyzer (M926, Sherwood Scientific Ltd., Cambridge, UK) as described in López-Climent et al. (2008) with some modifications. This methodology consisted in the extraction of 50 mg of fresh material by incubation in 5 ml of a buffer with 0.1 N HNO_3_ (Panreac, Barcelona, Spain) and 10% glacial acetic acid (Labbox Labware S.L., Barcelona, Spain) for 12 h at 25°C in darkness. After this period, 0.5 ml was taken for determinations in the chloride meter, using a commercial standard solution of 200 mg L^−1^ of Cl^−^ for the instrument calibration (Sherwood Scientific Ltd., Cambridge, UK).

### 2.4 Gas exchange and quantum yield parameters

Gas exchange parameters, including net photosynthesis rate (A), transpiration rate (E), and stomatal conductance (g_s_), were measured at 30 days of stress with a portable photosynthesis system (LI-6800, LI-COR Environmental, Lincoln, NE, USA) between 9 and 11 a.m. Light lamp was established at 1000 μmol m^−2^ s^−1^, air flow at 150 μmol mol^−1^, and CO_2_ of reference at 400 ppm. Three undamaged mature leaves from three randomly chosen plants were measured in each group, obtaining four measures per leaf after instrument stabilization at 1–2 min (Zandalinas et al., 2016).

In parallel, at each sampling point (10, 20, and 30 days of stress), photosystem II quantum yield (Φ_PSII_) was measured with a portable fluorometer (FluorPen FP-MAX 100, Photon Systems Instruments, Drasov Czech Republic) between 9 and 11 a.m. in nine light-adapted undamaged leaves from three different plants (Zandalinas et al., 2016).

### 2.5 Stomatal density

Stomata preparation was achieved by the obtaining of imprints with the help of dental adhesive resin (Aquasil Ultra+ Smart Wetting Impression Material, Dentsply Sirona, York, PA, USA) from the abaxial epidermis from mature full expanded leaves, taking the middle part between the midrib and the leaf edge, which has been demonstrated as a representative part of the whole leaf (Geisler et al., 2000; Beaulieu et al., 2008). After this, transparent nail polish was distributed over the leaf mold, and carefully removed after 5 min, placing it in microscopy slides (Beaulieu et al., 2008). Stomata preparations were viewed in the optical microscope (Nikon Eclipse 80i microscope equipped with Nikon DXM1200F digital camera, Nikon, Tokyo, Japan), and the obtained images were used for counting the stomatal density.

### 2.6 Phytohormone analysis

Analysis of leaf and root content of the phytohormones ABA, SA, JA, and IAA was conducted as described in Durgbanshi et al. (2005), involving the extraction of 200 mg of freshly ground material in 2 ml of deionized water with a mill ball equipment (MillMix 20, Domel Železniki, Slovenija), adding 25 ng of [^2^H_6_]-ABA, [^13^C_6_]-SA, dehydro-jasmonic acid (DHJA), and 2.5 ng [^2^H_5_]-IAA as internal standards. After this, samples were centrifuged, the supernatant was collected, and the pH was adjusted between 2.8 and 3.2. After this, a double liquid:liquid partition with diethyl ether (Fisher Scientific, Hampton, NH, USA) was performed, recovering the organic phase, which was dried in a vacuum centrifuge evaporator (Speed Vac, Jouan, Saint Herblain Cedex, France). Finally, the dried pellet was resuspended in 0.5 ml of 90:10 (v:v) water:methanol through sonication (Elma S30) for 10 min, and filtered through 0.22-µm PTFE syringe filters. The filtrate was diluted 1:3 (v:v) with 90:10 (v:v) water:methanol and transferred to glass liquid chromatography vials.

Processed samples were injected to the UPLC-MS system, consisting of a UPLC equipment connected to a triple quadrupole through an orthogonal Z-spray interface (Xevo TQ-S, Waters Corp., Milford, MA, USA). A sample volume of 15 μl was injected in the equipment and separated at 40°C through a reversed-phase C_18_ column (50 × 2.1 mm, 1.6 µm particle size, Luna Omega, Phenomenex, Torrance, CA, USA), using a gradient of ultrapure water and acetonitrile, both supplemented with 0.1% formic acid, with a constant flow rate of 300 µl min^−1^ (**Supplementary Table 1**). The mass spectrometer gas flow was fixed at 250 L h^−1^, with a desolvation gas flow of 1,200 L h^−1^ at 650°C in multiple reaction monitoring (MRM) mode. The transitions and retention times used for phytohormone quantification are provided in **Supplementary Table 2**. Quantification was achieved through the injection of a standard curve prepared with commercial standards, using the internal standards mentioned above, and processed with the software MassLynx V4.2 (Waters Corp., Milford, MA, USA).

### 2.7 Gene expression analysis

RNA was extracted from fresh frozen tissue ground to fine powder with the RNeasy extraction kit from Qiagen according to the manufacturer’s instructions (Qiagen, Hilden, Germany), and the quality of the extracted RNA was measured with a Nanodrop spectrophotometer (Nanodrop 2000, Thermo Scientific, Wilmington, DE, USA) to determine the RNA concentration and the absorbance ratios 260/280 and 260/230 nm to check for contaminations or impurities. A total amount of 5 μg from the extracted RNA was treated with DNase to remove the possibly extracted DNA (DNase I, Fermentas, Waltham, MA, USA), again measuring the quality with the Nanodrop spectrophotometer. Finally, a total amount of 1 μg of the extracted RNA was retrotranscribed to cDNA using the Primescript RT Reagent Kit (Takara, Shiga, Japan). Target gene accessions were obtained by searching the Arabidopsis TAIR database (Berardini et al., 2015) to obtain the protein sequence, which was used to perform a TBLASTN in the Citrus sinensis genome v1.1 from Phytozome (Goodstein et al., 2012), finally obtaining the CDS sequence used for the primer design. The primers used for the analysis of gene expression are provided in **Supplementary Table 3**. Actin (ACT) and tubulin (TUB) were used as housekeeping genes to normalize gene expression levels (Vives-Peris et al., 2018b).

The RT-qPCR analysis was performed to analyze gene expression in an ABI Step One detection system (Applied Biosystems, Foster City, CA, USA). Briefly, the amplification was conducted in reactions containing 1 μl of cDNA solution, 5 μl of Maxima SYBR Green/ROX qPCR mix (Thermo Scientific, Wilmington, DE, USA), 1 μl of a 10 μM mix of forward and reverse primers (**Supplementary Table 3**), and 3 μl of sterile deionized water to achieve a final volume of 10 μl per reaction. The amplification curve of temperatures consisted in 10 min at 95°C for preincubation and 40 cycles of amplification (each one with 10 s at 95°C for denaturation followed by 10 s at 60°C for annealing and an extension of 20 s at 72°C). The obtained results were processed with StepOne Software v2.3 and Relative Expression Software Tool v2 (REST; Pfaffl, 2001; Pfaffl et al., 2002).

### 2.8 Statistical analysis

Statistical analysis was performed with Infostat 2020 software (Universidad Nacional de Córdoba, Córdoba, Argentina). Data were subjected to one- or two-way analysis of variance (ANOVA) to compare the data between the four rootstock/scion combination and a Tukey *post-hoc* test (*p* ≤ 0.05) to compare the data obtained from stressed plants with their respective control. Microsoft Excel 365 (Microsoft, Albuquerque, NM, USA) was used for the representation of column graphs, whereas Sigmaplot v14.0 (Systat Software, Chicago, IL, USA) was used for representing radar plots and the principal component analysis (PCA). This statistical method consists in the description of the variation from *p* random variables in new uncorrelated ones (principal components) and ordering them by the percentage of the total variation explained by each one (Pearson, 1901; Hotelling, 1933).

## 3 Results

### 3.1 Plant phenotype

After 30 days of stress, mild phenotypic damage was observed in salt-stressed plants, but only in those plants grafted onto CC. In plants of both rootstock/scion combinations, CC-NA and CC-OR, some leaves (<5% of total leaves) were slightly curved under salt stress conditions (**Supplementary Figure 2**). In addition to the leaf phenotype, total fresh biomass was also evaluated, but no significant differences were appreciated among the different groups (**Supplementary Table 4**).

### 3.2 Malondialdehyde and proline content

**Figure 1:**
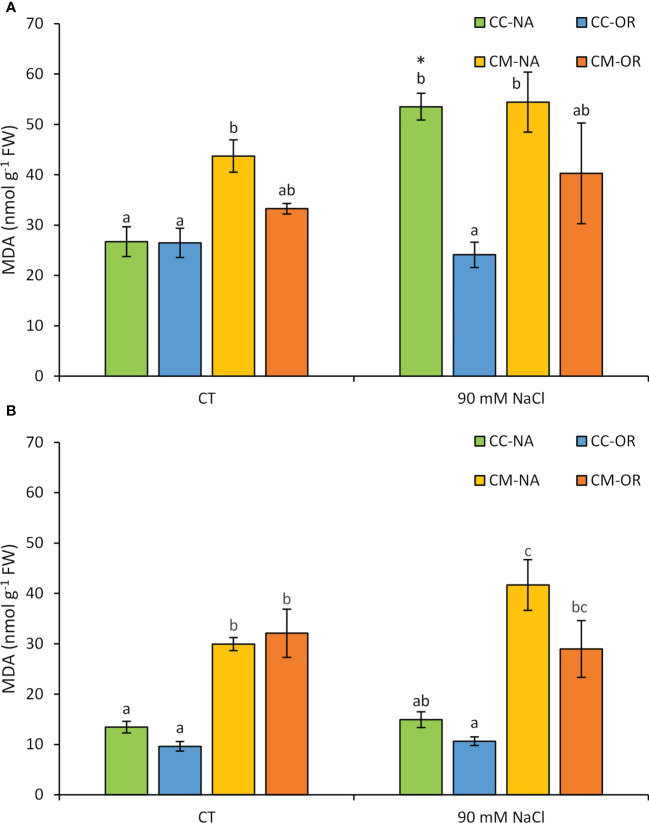
Leaf **(A)** and root **(B)** malondialdehyde content. Malondialdehyde content in leaves and roots of control and salt-stressed plants of CC-NA (green bars), CC-OR (blue bars), CM-NA (yellow bars), and CM-OR (orange bars) after 30 days. Values are the mean of three replicates ± standard error. Asterisks denote statistically significant differences in the stressed plants related to control at *p* ≤ 0.05. Different letters denote statistically significant differences among the rootstock/scion combinations for each treatment at *p* ≤ 0.05.

MDA concentration was evaluated in leaves and roots after 30 days of salt stress (**Figure 1**). Only plants with the rootstock/scion combination CC-NA exhibited a significant increase of MDA leaf content as a consequence of salt stress, with values 2.0-fold higher than those determined in leaves from CC-NA non-stressed plants (**Figure 1A**). On the other hand, salt treatment did not affect root MDA content, but higher values were observed in roots of plants grafted onto CM in both control and salt stress conditions, yielding a 2.8-fold mean increase in roots of CM-grafted plants in comparison to CC-grafted plants, regardless of the salt stress treatment (**Figure 1B**).

Meanwhile, proline content did not exhibit statistically significant differences neither in leaves nor in roots after 30 days, depending on neither the rootstock/scion combination nor the salt stress treatment (**Supplementary Figure 3**).

### 3.3 Chloride and sodium accumulation

**Figure 2:**
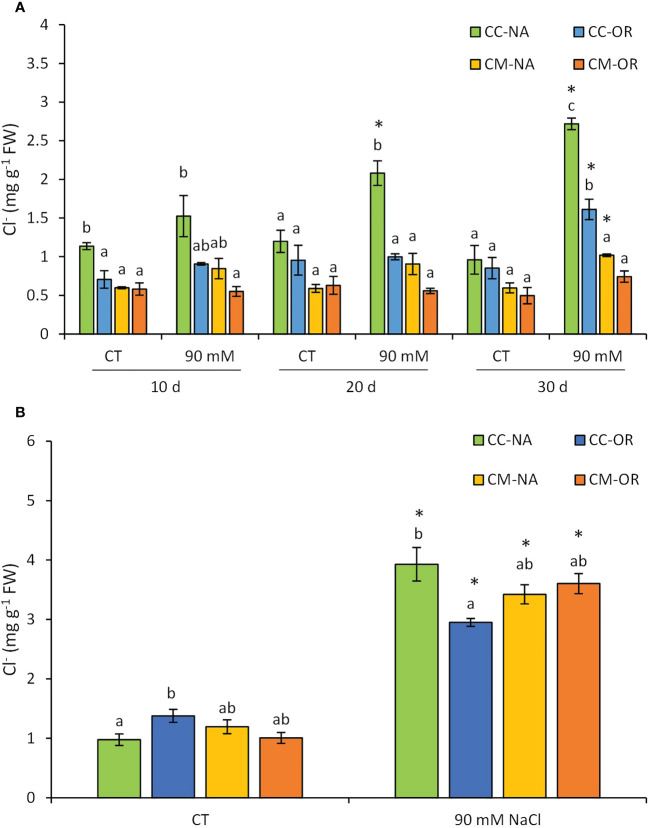
Leaf **(A)** and root **(B)** chloride content. Chloride ion content in leaves and roots of control and salt-stressed plants of CC-NA (green bars), CC-OR (blue bars), CM-NA (yellow bars), and CM-OR (orange bars). Values are the mean of three replicates ± standard error. Asterisks denote statistically significant differences in the stressed plants related to control at *p* ≤ 0.05. Different letters denote statistically significant differences among the rootstock/scion combinations for each treatment at *p* ≤ 0.05.

The accumulation of Cl^−^ varied among groups of plants, considering both salt stress treatment and rootstock/scion combination, and in both organs, leaves and roots (**Figure 2**). In leaves (**Figure 2A**), CC-NA plants were the fastest in the accumulation of Cl^−^ ions, being the only one that exhibited differences related to their control at 20 days (1.8-fold increase). This difference increased at 30 days, reaching values 2.8-fold higher in salt-stressed plants. However, at 30 days, CC-OR and CM-NA salt-stressed plants accumulated higher amounts of Cl^−^ ions as well (89.1% and 70.1% higher than controls, respectively), whereas leaves of CM-OR stressed plants did not show an increase of Cl^−^ content in comparison to control. Meanwhile, after 30 days of stress, root Cl^−^ content (**Figure 2B**) increased in salt-stressed plants in all rootstock/scion combinations in comparison to their respective controls, with the highest accumulation observed in CC-NA salt-stressed plants (4.4-fold increase).

In parallel to chloride, sodium leaf content exclusively increased in plants grafted onto CC after 30 days, with levels 4.35 and 4.47 times higher than those in the non-stressed CC-NA and CC-OR plants, respectively (**Supplementary Table 5**). After 30 days from the stress onset, Na^+^ root content also increased independently of the rootstock/scion combination, with values in salt-stressed plants approximately four times higher than their respective controls (**Supplementary Table 5**).

### 3.4 Gas exchange, chlorophyll fluorescence parameters, and stomatal density

**Figure 3:**
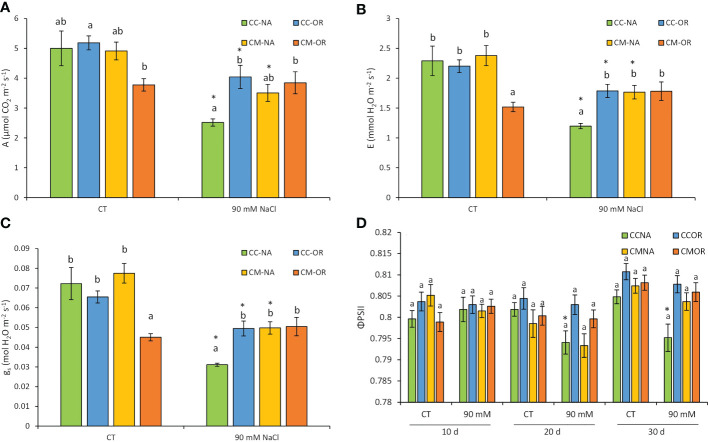
Photosynthesis-related parameters. Gas exchange parameters of leaves from control and salt-stressed plants of CC-NA (green bars), CC-OR (blue bars), CM-NA (yellow bars), and CM-OR (orange bars). **(A)** Net photosynthetic rate at 30 days. **(B)** Transpiration at 30 days. **(C)** Stomatal conductance at 30 days. **(D)** Quantum yield at 10, 20, and 30 days. Values are the mean of the data obtained from three leaves with four measurements per leaf ± standard error. Asterisks denote statistically significant differences in the stressed plants related to control at *p* ≤ 0.05. Different letters denote statistically significant differences among the rootstock/scion combinations for each treatment at *p* ≤ 0.05.

Plants from CM-OR combination exhibited the lowest values of A, E, and g_s_ under control conditions, but this situation was reverted under 30 days of salt stress, when CC-NA plants exhibited high decreases of these three parameters (**Figure 3**). As a result of salt stress treatment, A decreased in plants from all rootstock/scion combinations except for CM-OR (49.7%, 22.0%, and 28.6% in CC-NA, CC-OR, and CM-NA, respectively, **Figure 3A**). Similarly, E and g_s_ levels were reduced after 30 days of salt stress treatment in all plant combinations except CM-OR, with this decrease being more evident in CC-NA plants (reductions of approximately 50% in both parameters, **Figures 3B, C**).

As shown in **Figure 3D**, only plants with the combination CC-NA had significant reductions in quantum yield of photosystem II (Φ_PSII_) under stress (1.0% and 1.2% with respect to control at 20 and 30 days, respectively), with this parameter reflecting the efficiency of photosystem II, a key element for the light energy absorption, which is usually damaged under stressful conditions. Salt-stressed plants from the other rootstock/scion combinations (CC-OR, CM-NA, and CM-OR) did not exhibit variations in this parameter during all the experiments in comparison to their respective controls.

**Figure 4:**
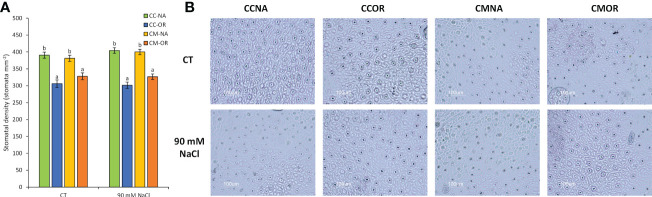
Stomata-related parameters. **(A)** Stomatal density in leaves of CC-NA (green bars), CC-OR (blue bars), CM-NA (yellow bars), and CM-OR (orange bars) control and salt-stressed plants. **(B)** Microscope photographs from stomata preparations from leaves of control and salt-stressed plants after 30 days. Values are the mean of 20 leaves ± standard error. Different letters denote statistically significant differences among the rootstock/scion combinations for each treatment at *p* ≤ 0.05.

A scion-dependent effect was detected in the stomatal density, since combinations with OR as the aerial tissues exhibited a mean diminution of stomatal density of 19.9% in comparison to those plants grafted with NA, independently of the salt stress treatment (**Figure 4**).

### 3.5 Chlorophyll and carotenoid content

Slight differences were observed in chlorophyll and carotenoid leaf content between control and salt-stressed plants after 30 days (**Supplementary Figure 4**). Under stress, Chl_b_ and total chlorophyll content increased exclusively in leaves of CC-NA plants. Additionally, contrasting concentrations of Chl_a_ were observed in stressed plants grafted onto CC, with values 20.7% higher in CC-NA leaves in comparison to CC-OR. No differences in the total carotenoid content were caused by salt stress treatment.

### 3.6 Phytohormone content

**Figure 5:**
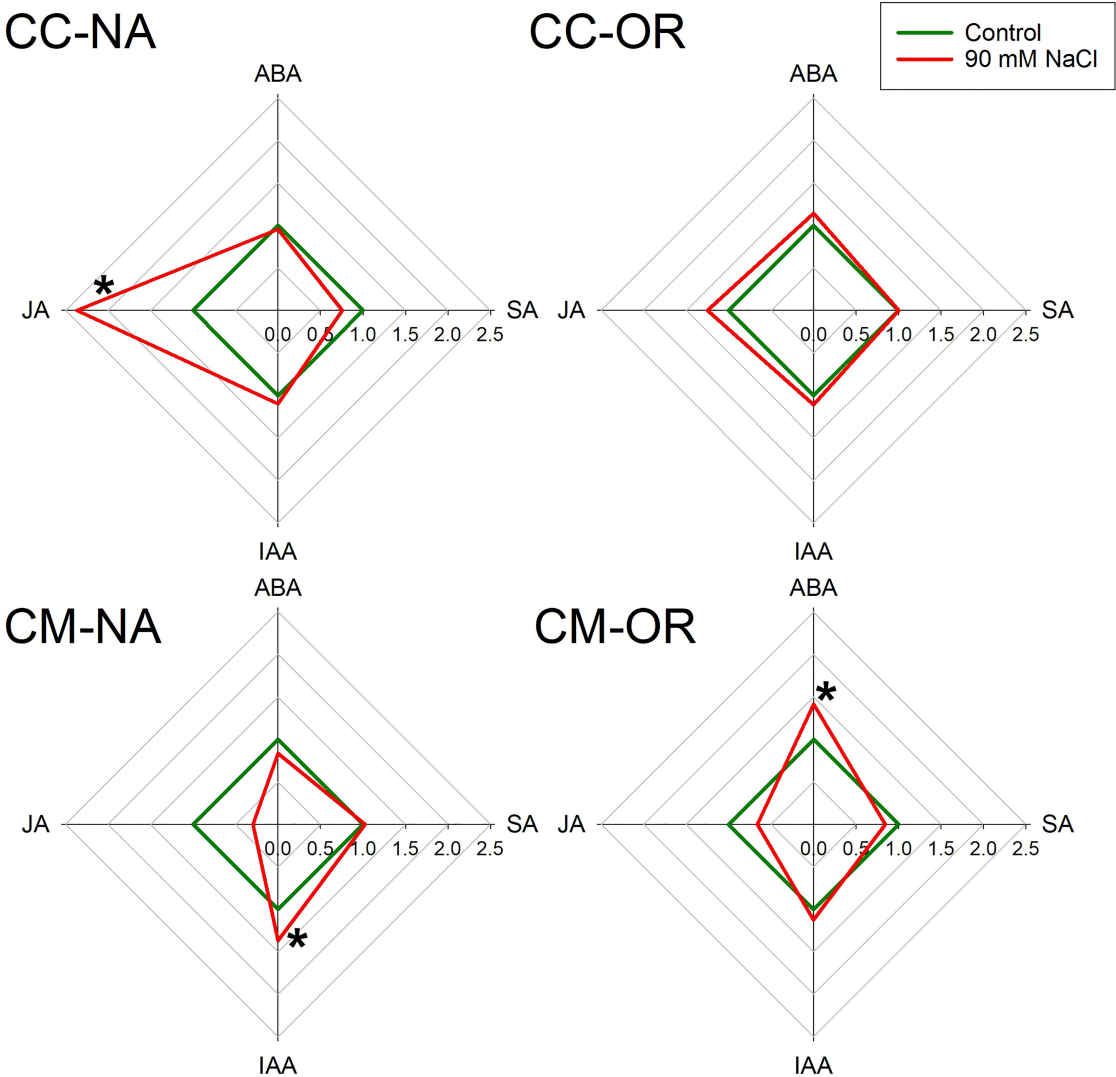
Leaf phytohormone content. Radar plots with leaf relative content of abscisic acid (ABA), jasmonic acid (JA), salicylic acid (SA), and indole acetic acid (IAA) in control (green lines), and 90 mM NaCl stressed plants (red lines) of CC-NA, CC-OR, CM-NA, and CM-OR after 30 days. Data are mean of three replicates. Asterisks denote statistically significant differences between control and salt-stressed plants of each scion/rootstock combination at *p* ≤ 0.05.

**Figure 6:**
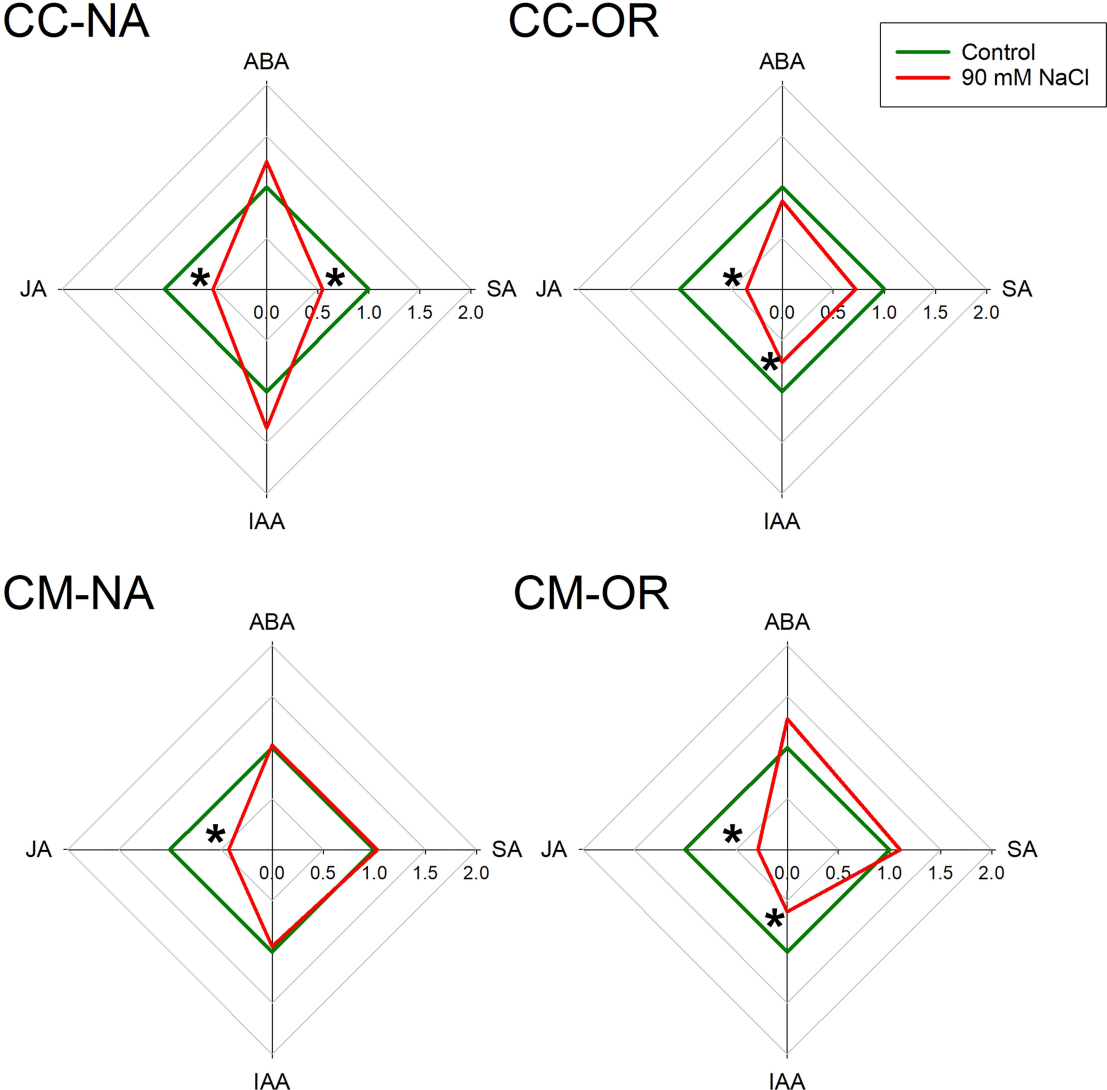
Root phytohormone content. Radar plots with root relative content of abscisic acid (ABA), jasmonic acid (JA), salicylic acid (SA), and indole acetic acid (IAA) in control (green lines) and 90 mM NaCl stressed plants (red lines) of CC-NA, CC-OR, CM-NA, and CM-OR after 30 days. Data are mean of three replicates. Asterisks denote statistically significant differences between control and salt-stressed plants of each scion/rootstock combination at *p* ≤ 0.05.

After 30 days of salt stress treatment, leaf and root phytohormone content was differentially affected depending on the rootstock/scion combination (**Figures 5**, **6**; **Supplementary Table 6**). Only plants with CM-OR combination increased leaf ABA content after this period (1.41-fold higher than its respective control), whereas no differences were appreciated in the root content of this plant hormone between control and salt-stressed plants of any rootstock/scion combination (**Figures 5**, **6**). **Figures 5**, **6** show that JA accumulation pattern was contrasting between leaves and roots, with increased concentrations in CC-NA leaves (values 2.4 times higher than control), but decreased concentrations in roots (47.4% and 71.3%, in CC-NA and CM-OR plants, respectively). Meanwhile, salt stress only caused a decline in SA content in CC-NA plants (24.5% and 45.3% of reduction in leaves and roots with respect to non-stressed plants). Finally, the leaf content of the auxin IAA only varied in CM-NA plants watered with NaCl supplemented solution, which showed concentrations 36.7% higher than control (**Figure 5**), while in roots, its content decreased in the combinations that had OR as aerial tissues, with reductions of 28.5% and 39.2% in the root IAA content of CC-OR and CM-OR plants (**Figure 6**).

### 3.7 Principal component analysis

**Figure 7:**
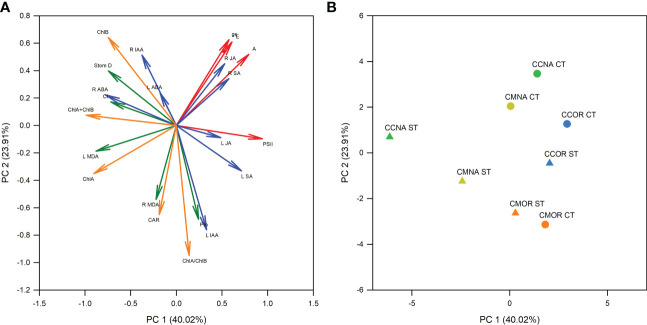
Principal component analysis. PCA with the physiological and biochemical data presented in this work. **(A)** Loading plot, representing photosynthesis related parameters (red arrows), phytohormone content (blue arrows), chlorophyll and carotenoid content (orange arrows), and other biochemical and physiological parameters (green arrows). **(B)** Scores plot, including the different groups of rootstock/scion combinations, CC-NA (green symbols), CC-OR (blue symbols), CM-NA (yellow symbols), and CM-OR (orange symbols) under control (circles) and salt stress (triangles) conditions.

Additionally, a PCA was performed in order to check the most important variables involved in the different tolerance of each rootstock/scion combination to salt stress after 30 days of 90 mM NaCl treatment (**Figure 7**). The first principal component (PC1) comprised 40.0% of the total variation and the second component (PC2) comprised 23.9%, as shown in the PCA graph with 63.9% of the variation. Additionally, the first component was the most important one separating the data obtained from control and salt-stressed groups of plants. According to the positioning in the principal components graph (**Figure 7B**), the rootstock/scion combination CC-NA (the most sensitive) showed the highest difference between control and stressed plants, whereas in the most tolerant one (CM-OR), this difference was the lowest.

### 3.8 Gene expression

**Figure 8:**
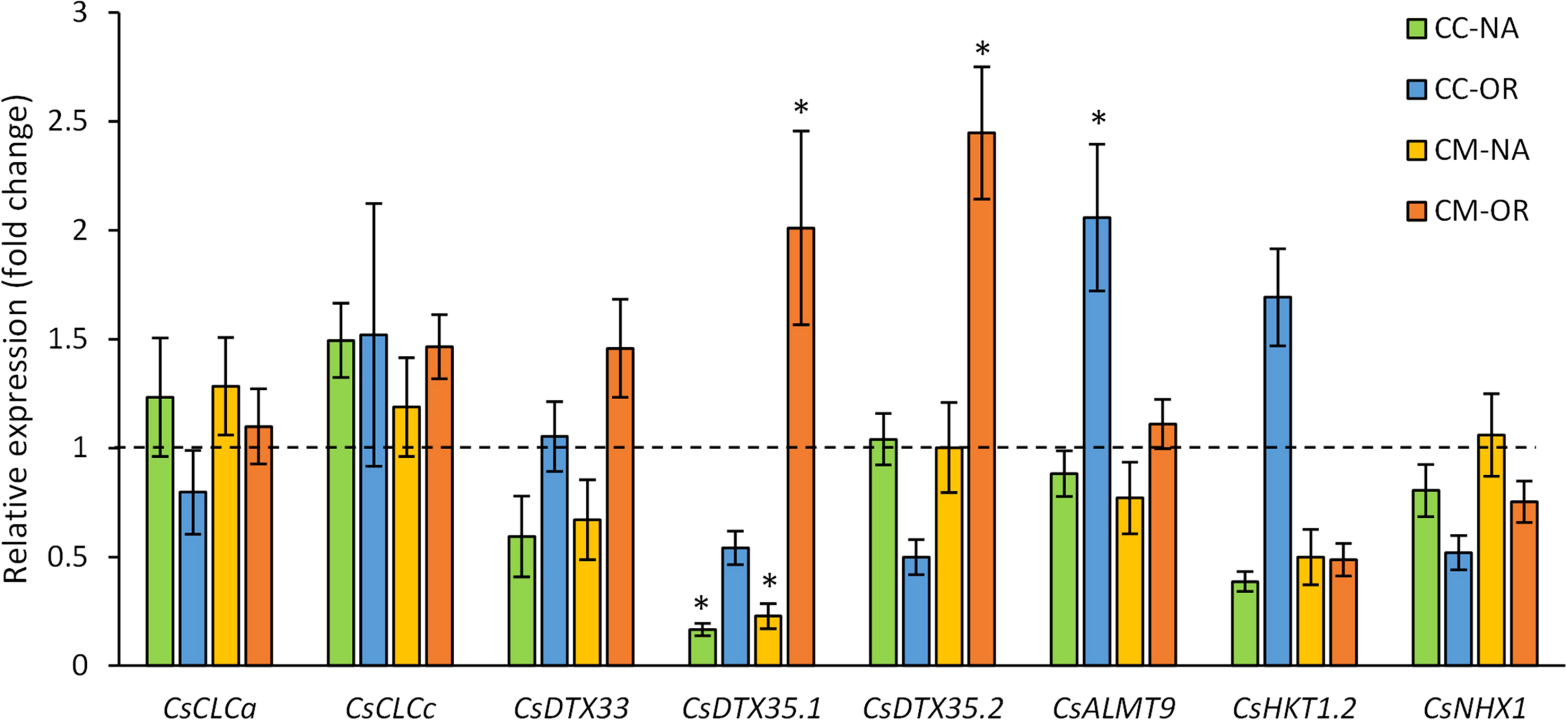
Gene expression. Relative expression of genes involved in Cl^−^ and Na^+^ uptake in the vacuoles in CC-NA (green bars), CC-OR (blue bars), CM-NA (yellow bars), and CM-OR (orange bars) in control and 90 mM NaCl stressed plants. Data are mean of three replicates ± standard error. Asterisks denote statistically significant differences between control and salt-stressed plants of each scion/rootstock combination at *p* ≤ 0.05.

The expression of genes codifying for tonoplast Cl^−^ and Na^+^ transporters was analyzed in leaf samples obtained from control and salt-stressed plants after 30 days of treatment (**Figure 8**). Thus, some of the genes related to Cl^−^ uptake-related transporters, including *CsCLCa*, *CsCLCc*, and *CsDTX33*, were not affected by salt stress (**Figure 8**). However, under salt stress conditions, *CsDTX35.1* was downregulated in CC-NA and CM-NA plants (reduction of 83.3% and 77.2% of their relative expression, respectively), but in CM-OR plants, its expression suffered an overregulation 2.0 times higher than control (**Figure 8**). Similarly, leaves of CM-OR plants also exhibited a 2.5-fold induction of the gene *CsDTX35.2* due to salt stress, but a downregulation was recorded in CC-OR plants (50.0% related to control; **Figure 8**). This downregulation of *CsDTX35.2* in CC-OR plants subjected to salt stress was correlated with an induction of *CsALMT9* (2.1 times higher than controls, **Figure 8**).

The expression of the gene *CsHKT1.2*, codifying for a Na^+^ tonoplast transporter, was repressed in leaves from all the combinations except in CC-OR plants, with downregulation between 50.0% and 61.3% in salt-stressed plants in comparison to their respective controls (**Figure 8**). No differences were observed in the expression of the gene related to other studied Na^+^ transporters in vacuole membrane, *CsNHX1* (**Figure 8**).

## 4 Discussion

Citrus is one of the most relevant fruit crops worldwide, whose productivity is being limited by the effects of climate change. Among abiotic stresses affecting this crop, high salinity is one of the most important, since citrus are usually grown in coastal areas where salinization of irrigation water is a main concern. Traditionally, approaches such as the use of tolerant rootstocks or the application of additional water to dilute the salt bulk have been used for mitigating the deleterious effect of this harmful situation, but strategies focused on the use of tolerant scions are practically unexplored (Ziogas et al., 2021). Some authors have intended to develop a platform to screen the rapid tolerance of citrus varieties to salt stress by using excised young twigs without roots (Ben Yahmed et al., 2016), which does not seem to be a realistic approach since citrus are commercially cultured grafted onto different rootstocks.

In this work, a comparative analysis of the response of the four different citrus rootstock/scion combinations to salt stress was performed. To ensure that the observed responses were due to tolerance mechanisms and not to an excessive damage, a mild salt stress was applied, selecting a 90 mM NaCl concentration as used in previous works with citrus (Vives-Peris et al., 2018a), instead of higher concentrations used in other works, which could lead to an excessive damage not representing field situations. The low severity of the applied stress was supported by the small differences observed in the proline accumulation pattern, an amino acid that acts as a compatible osmolyte to reduce the osmotic stress induced by high salinity (**Supplementary Figure 3**; Vives-Peris et al., 2018a). Apart from its osmoprotectant role, other beneficial properties have been previously described for proline, including a high metal chelating capacity (Dar et al., 2016) and a role in the maintenance of the redox potential through reactive oxygen species (ROS) inactivation, since it is considered a non-enzymatic antioxidant (Kavi Kishor et al., 2022).

The maintenance of chlorophyll and carotenoid levels (**Supplementary Figure 4**) revealed a slight increase in Chl_b_ content in leaves of CC-NA plants, which could indicate a higher sensitivity of this genotype combination to the stress and a response to ameliorate the decrease in photosynthesis-related parameters. In fact, a similar increase in Chl_b_ concentration under mild salt stress conditions compared to control has been reported in lettuce plants subjected to 50 mM NaCl for 8 days, with its endogenous content being reduced in the presence of higher concentrations of NaCl (Shin et al., 2020). Chlorophyll and carotenoid contents have been described as markers of salt stress tolerance, with their levels usually decreased in sensitive plants subjected to this adverse condition (Ashraf and Harris, 2013). The few variations of their concentration observed in our work support the low severity of the applied stress conditions in both terms, NaCl concentration and duration.

The results obtained in this work provide evidence on some of the main scion-related morphological, physiological, and molecular mechanisms that are involved in citrus tolerance to high salinity in the irrigation solution. One of the most important factors contributing to this tolerance is the regulation of the photosynthetic apparatus. Thus, in a multitude of plant species, including citrus, the decrease in photosynthetic related parameters (including A, E, g_s_, and Φ_PSII_) under salt stress conditions has been widely studied, which are maintained when plants tolerate this adverse situation (Vives-Peris et al., 2018a), and their regulation may contribute to salt stress tolerance through the maintenance of the intrinsic water use efficiency (iWUE) levels, ensuring an optimal use of water and carbon (Fernández-García et al., 2014). This agrees with our results, since under salt stress, photosynthesis-related parameters decreased the most in CC-NA plants but were not affected in CM-OR plants (**Figure 3**). The decrease in stomatal conductance and transpiration is mainly, but not exclusively, mediated by ABA, and minimizes water loss and reduces the uptake of Na^+^ and Cl^−^ ions (Acosta-Motos et al., 2015). Moreover, Φ_PSII_ only decreased in CC-NA plants under stress (**Figure 3D**), confirming the importance of the maintenance and reparation of the photosynthetic system in citrus plants when they are subjected to abiotic stress conditions, as it has been recently described for other stresses such as drought, high light, high temperatures, and their combinations (Balfagón et al., 2022).

All these parameters are highly involved in stress tolerance, since the maintenance of the photosynthetic system is crucial in the defense against hazardous environmental conditions, and allow to point out that the tolerance of OR to salt stress is higher than that of NA. This, along with the higher oxidative damage in CC-NA plants subjected to salt stress, marked an increase in MDA content, a subproduct of membrane lipid peroxidation (**Figure 1**), which is in concordance with the higher sensitivity of CC to salt stress in comparison to CM (which is moderately tolerant), although MDA basal levels were higher in CM-grafted plants, maybe due to the enhanced antioxidant system of CC, as it has been previously reported even under *in vitro* conditions (Vives-Peris et al., 2017). This higher MDA content in roots and leaves of plants grafted onto CM could be due to other parameters differentially affecting the rootstocks, such as soil texture or temperature, since previous works have reported that they have different tolerance to other abiotic stress conditions, such as low temperatures (Primo-Capella et al., 2022). Additionally, the higher MDA content could also be considered as beneficial, since it has been proposed as a signaling molecule whose transient accumulation under abiotic conditions can improve plant antioxidant responses to abiotic stress conditions through the induction of the expression of ALDHs (reviewed in Morales and Munné-Bosch, 2019).

There are several factors that can contribute to the enhanced tolerance of OR to salt stress, including the lower stomatal density of this genotype (**Figure 4**), which would contribute to better control the transpiration and, consequently, the uptake of water and Na^+^ or Cl^−^ excess, as it is shown by the correlation among leaf Cl^−^ and Na^+^ levels found in the different genotypes (the lowest in the tolerant CM-OR and the highest and fastest in the sensitive CC-NA, **Figure 2**; **Supplementary Table 5**). Although Cl^−^ is an essential element for plants, involved in key processes such as photosynthesis, stomatal regulation, nitrogen metabolism, or osmoregulation (Colmenero-Flores et al., 2019), excessive levels of this ion as a consequence of soil salinity are the main factor determining leaf damage in citrus under salt stress in comparison with Na^+^ toxicity or the osmotic component (López-Climent et al., 2008). The improved photosynthetic regulation could be partially mediated by phytohormones such as ABA, whose content was only increased in leaves of CM-OR plants after 30 days of stress (**Figure 5**; **Supplementary Table 6**), this molecule being one of the main signals inducing the stomatal closure in plants cultured under several adverse conditions (Bharath et al., 2021). Although this phytohormone is often accumulated under salt stress conditions, in this work, no increase was observed in plants of remaining rootstock/scion combinations, which could confirm that the stress intensity was moderated. Thus, the increase in ABA content in CM-OR leaves was relatively low and could not be enough for an efficient control of stomatal closure, and consequently, this difference could be due to an ABA-independent stomatal control as described by previous works (Hsu et al., 2018), which could be regulated by other hormones such as SA or oxylipins (Montillet et al., 2013) or even calcium (Wang et al., 2012).

The accumulation of JA under abiotic stress conditions has been previously studied and is often accompanied by an SA content decrease as in this case; thus, both phytohormones have been usually reported as antagonists (Wang et al., 2021). In our work, a JA accumulation was observed in leaves of the sensitive combination CC-NA, while a decrease in leaf SA content was observed only in this combination. However, a general decrease in JA root levels was observed, which is more evident in those plants grafted onto the salt-tolerant rootstock CM. This would also support the idea of the incipient grade of salt stress at this point, since existing literature describes lower JA contents in plants tolerant to salt stress (Shahzad et al., 2015). It could also verify the higher tolerance of CM to high salinity with respect to CC. Interestingly, root IAA levels decreased in salinized plants having OR as scion. Therefore, the grafted scion would be essential for IAA synthesis and transport to the roots. Hence, low IAA translocation to roots could improve salt stress tolerance by the reduction of root branching, thus limiting the surface of Na^+^ or Cl^−^ absorption. In fact, the main biosynthesis route of this auxin derives from tryptophan, which is produced in the chloroplasts via the shikimate pathway, stressing the importance of the aerial tissues in the production of this phytohormone (Casanova-Sáez et al., 2021).

The accumulation of Cl^−^ and Na^+^ ions in the vacuole could be another strategy to reduce the negative impact of salt stress, as it has been previously described in other plant species such as Arabidopsis or soybean (reviewed in Wu and Li, 2019). Thus, several tonoplast transporters have been identified, including Cl^−^ transporters such as CLCa, CLCc, DTX33, DTX35, and ALMT9 (De Angeli et al., 2009; Baetz et al., 2016; Zhang et al., 2017) or Na^+^ transporters HKT1 or NHX1 (Martínez-Alcántara et al., 2015). The analyses of the expression of genes codifying for Cl^−^ or Na^+^ transporters resulted in an overexpression of *CsDTX35.1* and *CsDTX35.2* genes in the tolerant combination CM-OR (**Figure 8**). Consequently, these results suggest that *CsDTX35.1* and *CsDTX35.2* (two isoforms from *AtDTX35*) are crucial for Cl^−^ transport through the tonoplast in citrus, as it has been previously described for Arabidopsis (Zhang et al., 2017), whereas the role of CLC transporters would be less relevant since their gene expressions are not affected by salt stress. This information could confirm the enhanced capability of CM-OR plants to synthesize these transporters, responsible for the vacuole compartmentalization of Cl^−^ ions from the cytosol under salt stress conditions (considering that Cl^−^ is the most damaging component of salt stress in citrus) whereas Na^+^ transport through tonoplast could be less relevant (López-Climent et al., 2008). Thus, since Cl^−^ content was lower in CM-OR plants, a double strategy could be used in these plants, excluding ion root uptake and enhancing its compartmentalization in the vacuoles. In the sensitive plant combinations, higher levels of Cl^−^ ions would be dispersed in the cytosol or moved through xylem to other tissues following the transpiration stream (Geilfus, 2018). The identification of these candidate genes, *CsDTX35.1* and *CsDTX35.2*, can be useful for future breeding citrus programs, either by selecting new varieties with higher expression of these genes in response to high salinity, or by increasing their expression through genetic engineering techniques. In this sense, Arabidopsis plants overexpressing the gene codifying for the tonoplast Na^+^ transporter *At*NHX1 improve their tolerance to salt stress (Pabuayon et al., 2021).

## 5 Conclusion

In addition to the rootstock, scion plays an important role in salt stress tolerance in citrus. This work shows some of the main scion-related morphological, physiological, and molecular mechanisms that are involved in citrus tolerance to high salinity and can be the bases for future breeding programs and could also provide mechanisms for selecting the best rootstock–scion combination under each particular condition, contributing to a more productive and sustainable citrus industry.

## Data availability statement

The raw data supporting the conclusions of this article will be made available by the authors, without undue reservation.

## Author contributions

VV-P and ML-C performed all experiments, VV-P, AG-C and RP-C conceived the study and the experimental design and wrote the manuscript. VV-P and MM-S performed the sample and data analysis. AG-C and RP-C supervised the work and provided financial resources. All authors contributed to the article and approved the submitted version.
