# Supplementary material for: Morphological, physiological, and molecular scion traits are determinant for salt-stress tolerance of grafted citrus plants
Source: Front Plant Sci. 2023 Apr 20;14:1145625. doi: 10.3389/fpls.2023.1145625 (PMC10157061; doi:10.3389/fpls.2023.1145625)
Supplement: Supplementary file 5 [file Table_1.docx]

**Supplementary Table 1.** Mobile phase gradient used for the chromatographic separation of phytohormones. “A” refers to ultrapure water, and “B” to acetonitrile, both supplemented with 0.1% formic acid.

| **Time (min)** | **Flow (mL min^-1^)** | **%A** | **%B** |
| --- | --- | --- | --- |
| 0 | 0.3 | 90 | 10 |
| 2 | 0.3 | 90 | 10 |
| 6 | 0.3 | 10 | 90 |
| 7 | 0.3 | 90 | 10 |
