# Supplementary material for: Morphological, physiological, and molecular scion traits are determinant for salt-stress tolerance of grafted citrus plants
Source: Front Plant Sci. 2023 Apr 20;14:1145625. doi: 10.3389/fpls.2023.1145625 (PMC10157061; doi:10.3389/fpls.2023.1145625)
Supplement: Supplementary file 6 [file Table_2.docx]

**Supplementary Table 2.** Transitions and retention times used for phytohormone determination through UPLC-MS.

| **Compound** | **Use** | **Ionization mode** | **Transition** | **Cone Voltage (V)** | **Collision Energy (eV)** | **Retention time (min)** | **Internal standard** |
| --- | --- | --- | --- | --- | --- | --- | --- |
| ABA | Target | - | 263>153 | 25 | 12 | 4.40 | [^2^H_6_]-ABA |
| SA |  | - | 137>93 | 25 | 15 | 4.10 | [^13^C_6_]-SA |
| JA |  | - | 209>59 | 25 | 15 | 4.69 | DHJA |
| IAA |  | + | 176>130 | 25 | 15 | 4.11 | [^2^H_5_]-IAA |
| [^2^H_6_]-ABA | Internal standard | - | 269>159 | 25 | 12 | 4.40 | - |
| [^13^C_6_]-SA |  | - | 143>99 | 25 | 15 | 4.10 | - |
| DHJA |  | - | 211>59 | 25 | 15 | 4.93 | - |
| [^2^H_5_]-IAA |  | + | 179>135 | 25 | 15 | 4.09 | - |
