# Supplementary material for: Morphological, physiological, and molecular scion traits are determinant for salt-stress tolerance of grafted citrus plants
Source: Front Plant Sci. 2023 Apr 20;14:1145625. doi: 10.3389/fpls.2023.1145625 (PMC10157061; doi:10.3389/fpls.2023.1145625)
Supplement: Supplementary file 7 [file Table_3.docx]

**Supplementary Table 3.** List of primers used for gene expression analysis with RT-qPCR.

| **Gene** | **Accession** | **Forward primer** | **Reverse primer** | **Amplicon size (bp)** |
| --- | --- | --- | --- | --- |
| *CsACT* | orange1.1g037845m | CCCTTCCTCATGCCATTCTTC | CGGCTGTGGTGGTAAACATG | 105 |
| *CsTUB* | orange1.1g013335m | GGGGCAAAATGAGCACTAAA | CGCCTGAACATCTCCTGAAT | 187 |
| *CsCLCa* | orange1.1g003966m | CCAATCCAGTGGCAAGAGCA | GGAAACTTTGGCTCCAACAA | 149 |
| *CsCLCc* | orange1.1g003885m | GAATGGGATCAGGAACGTCA | CAACCTTCCGTCTTGCTCTC | 183 |
| *CsDTX33* | orange1.1g015884m | CTCCCTTCTTGGCATAACCA | CCCACAAATCATTCCAATCC | 199 |
| *CsDTX35.1* | orange1.1g035514m | GGTGCAGCTATTGCATACGA | CCAGACAAAGCATCACAGCT | 169 |
| *CsDTX35.2* | orange1.1g043161m | GGGCAGCATTCATTGAGATT | CAGCATAGCTTCCCAACCAT | 182 |
| *CsALMT9* | orange1.1g012114m | GGATTTAATCGGGGATTGGG | CATCGTCGGGTACAGTTTTG | 159 |
| *CsHKT1.2* | orange1.1g045632m | CATACGGGAATGTGGGATTC | GCTTTGCCACCTTTCATGTT | 181 |
| *CsNHX1* | orange1.1g009645m | GTTTCGACGCTTCTGAATGA | GCAGTACACAGGCCAAAAGC | 158 |
