# Supplementary material for: Morphological, physiological, and molecular scion traits are determinant for salt-stress tolerance of grafted citrus plants
Source: Front Plant Sci. 2023 Apr 20;14:1145625. doi: 10.3389/fpls.2023.1145625 (PMC10157061; doi:10.3389/fpls.2023.1145625)
Supplement: Supplementary file 8 [file Table_4.docx]

**Supplementary Table 4.** Total biomass after 30d of the stress onset. Asterisks denote significant differences in the stressed plants related to control at P ≤ 0.05. Different letters denote significant differences among the rootstock/scion combinations for each treatment at P ≤ 0.05.

|  | **Fresh weight (g)** | |
| --- | --- | --- |
|  | **Control** | **90 mM NaCl** |
| CC-NA | 356.8±40.2^a^ | 338.0±25.1^a^ |
| CC-OR | 327.2±31.1^a^ | 317.6±21.8^a^ |
| CM-NA | 345.6±25.7^a^ | 325.4±46.2^a^ |
| CM-OR | 314.4±43.3^a^ | 317.1±33.4^a^ |
