# Supplementary material for: Morphological, physiological, and molecular scion traits are determinant for salt-stress tolerance of grafted citrus plants
Source: Front Plant Sci. 2023 Apr 20;14:1145625. doi: 10.3389/fpls.2023.1145625 (PMC10157061; doi:10.3389/fpls.2023.1145625)
Supplement: Supplementary file 9 [file Table_5.docx]

**Supplementary Table 5.** Leaf and root sodium content in control and 90 mM NaCl stressed plants after 30d. Asterisks denote statistically significant differences in the stressed plants related to control at P ≤ 0.05. Different letters denote statistically significant differences among the rootstock/scion combinations for each treatment at P ≤ 0.05. Na+ content was determined by the company Eurofins Agroambiental (Lleida, Spain) by inductively coupled plasma optical emission spectroscopy (ICP-OES) according to the stablished protocols (UNE-EN ISO/IEC 17025:2017).

|  | **Leaf Na^+^ content (mg kg^-1^ DW)** | | **Root Na^+^ content (mg kg^-1^ DW)** | |
| --- | --- | --- | --- | --- |
|  | **Control** | **90 mM NaCl** | **Control** | **90 mM NaCl** |
| CC-NA | 582.0±76.7^a^ | 2532.7±305.6^a*^ | 2835.3±221.1^a^ | 13597.6±251.9^a*^ |
| CC-OR | 463.3±120.2^a^ | 2072.7±357.2^a*^ | 2133.7±673.2^a^ | 13484.0±260.6^a*^ |
| CM-NA | 336.3±91.2^a^ | 926.7±244.6^a^ | 1993.3±210.9^a^ | 13540.0±234.7^a*^ |
| CM-OR | 497.3±72.7^a^ | 1670.0±513.4^a^ | 2455.3±441.5^a^ | 12991.3±722.9^a*^ |
