# Supplementary material for: Morphological, physiological, and molecular scion traits are determinant for salt-stress tolerance of grafted citrus plants
Source: Front Plant Sci. 2023 Apr 20;14:1145625. doi: 10.3389/fpls.2023.1145625 (PMC10157061; doi:10.3389/fpls.2023.1145625)
Supplement: Supplementary file 10 [file Table_6.docx]

**Supplementary Table 6.** Leaf and root phytohormone content in control and 90 mM NaCl stressed plants after 30d. Asterisks denote statistically significant differences in the stressed plants related to control at P ≤ 0.05. Different letters denote statistically significant differences among the rootstock/scion combinations for each treatment at P ≤ 0.05.

| ***Leaves*** | **ABA (ng g FW^-1^)** | | **JA (ng g FW^-1^)** | | **SA (ng g FW^-1^)** | | **IAA (ng g FW^-1^)** | |
| --- | --- | --- | --- | --- | --- | --- | --- | --- |
|  | **CT** | **90 mM** | **CT** | **90 mM** | **CT** | **90 mM** | **CT** | **90 mM** |
| CC-NA | 28.33±0.42^ab^ | 27.05±3.65^a^ | 5.85±0.55^a^ | 13.86±3.42^ab*^ | 165.62±10.86^a^ | 125.11±13.83^a*^ | 9.80±0.43^ab^ | 10.75±0.53^a^ |
| CC-OR | 24.33±2.32^ab^ | 27.81±2.83^a^ | 27.61±10.32^a^ | 34.45±12.31^b^ | 154.59±18.92^a^ | 154.86±21.33^a^ | 12.55±1.08^bc^ | 13.91±1.31^ab^ |
| CM-NA | 33.51±4.12^b^ | 27.99±2.71^a^ | 19.21±7.84^a^ | 5.70±0.79^a^ | 154.39±14.97^a^ | 157.99±10.96^a^ | 7.62±0.49^a^ | 10.41±0.46^a*^ |
| CM-OR | 22.61±2.72^a^ | 31.96±3.13^a*^ | 19.59±3.59^a^ | 12.97±3.29^ab^ | 185.32±36.01^a^ | 156.28±12.47^a^ | 14.47±0.95^c^ | 16.27±1.11^b^ |
|  | | | | | | | | |
| ***Roots*** | **ABA (ng g FW^-1^)** | | **JA (ng g FW^-1^)** | | **SA (ng g FW^-1^)** | | **IAA (ng g FW^-1^)** | |
|  | **CT** | **90 mM** | **CT** | **90 mM** | **CT** | **90 mM** | **CT** | **90 mM** |
| CC-NA | 5.08±0.26^a^ | 6.35±1.05^a^ | 27.32±2.03^ab^ | 14.37±1.81^b*^ | 152.17±35.12^a^ | 83.21±6.11^a*^ | 12.03±0.43^b^ | 16.35±2.48^b^ |
| CC-OR | 5.09±0.94^a^ | 4.40±0.38^a^ | 39.71±10.42^b^ | 13.85±3.01^ab*^ | 144.09±34.97^a^ | 103.61±8.21^a^ | 12.19±0.80^b^ | 8.72±0.39^a*^ |
| CM-NA | 6.23±0.83^a^ | 6.39±1.65^a^ | 14.37±2.76^a^ | 6.11±1.54^a*^ | 93.22±6.50^a^ | 95.92±11.95^a^ | 7.11±0.59^a^ | 6.74±0.74^a^ |
| CM-OR | 4.34±0.57^a^ | 5.56±0.86^a^ | 22.78±5.49^ab^ | 6.54±1.41^a*^ | 105.72±17.94^a^ | 116.73±6.29^a^ | 8.56±0.58^a^ | 5.21±0.29^a*^ |
